# Facile Synthesis of Pd Nanocubes with Assistant of Iodide and Investigation of Their Electrocatalytic Performances Towards Formic Acid Oxidation

**DOI:** 10.3390/nano9030375

**Published:** 2019-03-05

**Authors:** Xuan Liu, Zichao Li, Kuankuan Wang, Luming Zhou, Xihui Zhao, Wenhai Jiang, Qun Li, Yujia Deng

**Affiliations:** 1 School of Chemistry and Chemical Engineering, Qingdao University, Qingdao 266071, China; liuxuan2795@163.com (X.L.); zichaoli@qdu.edu.cn (Z.L.); wang13460062584@163.com (K.W.); 2018025207@qdu.edu.cn (L.Z.); zhaoxihui@qdu.edu.cn (X.Z.); jwh1785474676@163.com (W.J.); qunli501@163.com (Q.L.); 2 College of Life Sciences, Qingdao University, Qingdao 266071, China

**Keywords:** Pd nanocubes, shape-controlled, iodide, electrocatalytic, formic acid oxidation

## Abstract

This article presents a facile, one-pot method using the aqueous phase for the synthesis of high-quality Pd nanocubes. In this study, Pd chloride was used as the precursor, sodium iodide as capping agent, and poly(vinylpyrrolidone) as surfactant and reducing agent. The effects of different halogens on the morphology of Pd nanocrystals were investigated. The results showed that, in this synthesis system, the selection and proper amount of sodium iodide was essential to the preparation of high-quality Pd nanocubes. When iodide was replaced by other halogens (such as bromide and chloride), Pd nanocrystals with cubic morphology could not be obtained. In addition, we have found that NaBH_4_ can be used to efficiently remove inorganic covers, such as iodide, from the surface of Pd nanoparticles as synthesized. The Pd nanoparticles obtained were employed as electro-catalysts for formic acid oxidation, and they exhibited excellent catalytic activity and good stability towards this reaction.

## 1. Introduction

Pd nanomaterials are widely used as catalysts and have attracted much attention in recent years because of their excellent catalytic performances in chemical industry, energy science, and related fields [1,2,3,4]. Similar to many other catalysts, the catalytic activity and selectivity of Pd nanoparticles are strongly related to their size and surface structure. The catalytic performance of Pd nanoparticles can be optimized by adjusting their size and morphology, therefore reducing the amount of this noble metal catalyst in various catalytic reactions as much as possible. Recently, significant progress has been made, using various methods, for shape-controlled syntheses of Pd nanoparticles, such as cube, octahedron, dodecahedron, concave, flake, rod, and linear Pd nanoparticles [5,6,7,8,9,10,11,12,13,14,15,16,17,18,19,20,21]. Among the methods used to create shape-controlled syntheses of precious metal nanoparticles, one efficient strategy includes the introduction of small adsorbents, such as halogen ions, to selectively adsorb on specific crystal planes to change crystal growth. For example, Huang et al. reported a method of synthesizing high-quality Pd nanocubes by employing *N*,*N*-dimethylformamide (DMF) as solvent, sodium iodide as capping agent, poly (vinylpyrrolidone) (PVP) as surfactant, and palladium acetylacetonate as precursor. They found that the choice of iodide ion was key to the successful synthesis of single-crystalline Pd nanoparticles. When iodine ions were replaced by other halogen ions, polycrystalline Pd nanoparticles with no specific shapes were obtained [22]. In direct formic acid fuel cells, formic acid oxidation is the anode reaction, and Pd nanoparticles can catalyze the conversion of formic acid directly to carbon dioxide, exhibiting significantly high power density. In comparison with platinum nanomaterials, Pd nanomaterials have demonstrated superior catalytic performances towards formic acid oxidation [23,24,25]. Xia et al. reported a method to control the morphology and size of Pd nanoparticles through seed growth; using this method, Pd polyhedron nanoparticles such as chamfered cube, cubic octahedron, chamfered octahedron, and octahedron were successfully synthesized. Their study found that the (100) and (111) planes exposed to the Pd nanocrystals as prepared were different in proportion and exhibited different activities in catalyzing formic acid oxidation; the peak current density of nanocubes with fully exposed (100) facets was much higher than that of nano octahedrons [6]. Accordingly, we explored a simple, clean, and environmentally friendly method, such as using a water-based system, to synthesize high-quality Pd nanocubes as catalysts for formic acid oxidation reaction.

Herein, we report a facile method to effect a shape-controlled synthesis of Pd nanocubes using an aqueous phase system in the presence of PVP and sodium iodide. In this simple synthesis system, an iodine ion acted as the shape-controlled agent to induce the formation of cubic morphology of Pd nanoparticles, and PVP played the role of surfactant, stabilizer, and reductant. To guarantee the catalytic active sites on the surface of the Pd nanoparticles as synthesized, the surfactants and capping agents that adsorbed on the surface of Pd nanoparticles should be removed. In this work, we have also tested and demonstrated that NaBH_4_ can be effectively used to remove inorganic covers (Cl^−^, Br^−^, I^−^) and organic stabilizers (PVP) from the surface of Pd nanoparticles [26]. The Pd nanocubes obtained by this method showed excellent catalytic performance in formic acid oxidation.

## 2. Experimental Section

### 2.1. Chemicals and Materials

Pd (Ⅱ) chloride (PdCl_2_), Sodium iodide (NaI, GR.) were purchased from Shanghai Macklin Biochemical Co. Ltd (Shanghai, China). Poly(vinylpyrrolidone) (PVP K30, Mr ≈ 10000), Potassium bromide (KBr, GR.), potassium chloride (KCl, GR.), Sodium borohydride (NaBH_4_, GR.) were purchased from Sinopharm Chemical Reagent Co. Ltd (Shanghai, China). All reagents were used as received without further purification, and the water used in all experiments was ultrapure (Millipore water, 18.2 MΩ cm). The morphology and structure of the nanoparticles were characterized by scanning electron microscopy (SEM, Hitachi S-4800, Tokyo, Japan), and transmission electron microscopy (TEM, JEM-2100 at 200 kV, JEOL Ltd, Tokyo, Japan).

### 2.2. Synthesis of Pd Nanocubes

In a typical synthesis, 20 mg of PdCl_2_, 300 mg of NaI and 400 mg of PVP were added to 8.0 mL of ultrapure water, then stirred for 3 min at room temperature. The resulting homogeneous dark red solution was transferred to a 25 mL Teflon-lined, stainless-steel autoclave. The sealed vessel was then held at 200 °C for 6 h before it was cooled to room temperature. The heating rate of the autoclave was 6 °C min^−1^. The products were separated via centrifugation at 11,000 rpm for 20 min and further purified by ethanol two times and ultrapure water three times.

### 2.3. Removal of Stabilizer and Capping Agent from Pd Nanoparticles As-Synthesized

The preliminary washed Pd nanoparticles were dispersed in a 20 mL reaction flask containing 10 mL of deionized water. 60 mg of sodium borohydride was added, the cap was tightened, and it was sonicated for 30 min at room temperature. Subsequently, the reaction flask was placed in a constant temperature water bath, heated, and stirred at 85 °C for 8 h, and the solution in the reaction flask was then centrifuged at 11,000 rpm for 20 min and the supernatant was decanted. Finally, the products were dispersed in 2 mL ultrapure water for use.

### 2.4. Electrochemical Measurements

Electrochemical experiments were performed using a conventional three-electrode cell at room temperature, including a saturated calomel electrode (SCE) as the reference electrode, a Pt wire as the counter electrode, and a glassy carbon (GC) electrode (3 mm diameter) as the working electrode. All of the electrode potentials in this article were quoted versus the SCE. GC electrode was polished with Al_2_O_3_ Polishing powder (1.0 µm) for 15 min and washed with ultrapure water before electrochemical experiment. Subsequently, 8 µL of the water dispersion of Pd nanocrystals were dropped onto the GC electrode and dried at room temperature. The electrolyte for formic acid oxidation was a 0.25 M HClO_4_ + 0.25 M HCOOH solution, and the potential was scanned from −0.21 V to 0.95 V at a sweep rate of 50 mV s^−1^. Current density was obtained by normalizing the oxidation current to the electrochemical surface area (ECSA) of Pd nanocatalysts. The electrochemically active surface area (ECSA) was measured using hydrogen under potential deposition (Hupd) on Pd nanocubes. The cyclic voltammograms (CVs) were recorded in nitrogen-saturated 0.1 M HClO_4_ solution, and the potential was scanned from −0.215 V to 0.75 V at a sweep rate of 50 mV s^−1^ for 10 cycles to obtain the stable CVs.

## 3. Results and Discussion

**Figure 1:**
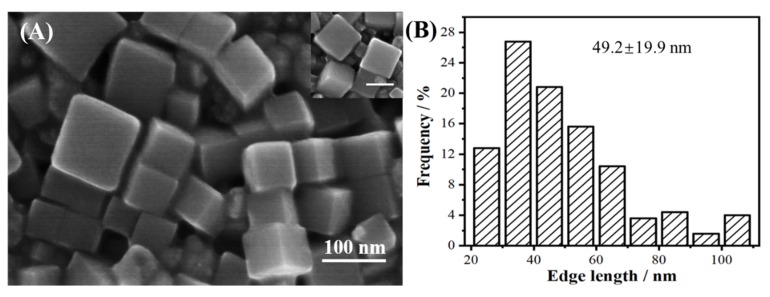
(**A**) Overview SEM image of the Pd nanocubes prepared using the standard synthesis. The inset shows the high-magnification SEM image of the Pd nanocubes, with a scale bar of 100 nm. (**B**) Histogram of edge-length of the Pd nanocubes.

Figure 1A showed a representative overview SEM image of the Pd nanocubes as synthesized. From the low-magnification SEM image, a successfully obtained Pd nanoparticles with complete cubic shape can be seen; from the high-magnification SEM image of the Pd nanocubes, the perfect cube shape of the Pd nanoparticles also can be seen (inset in Figure 1A). The yield of the Pd nanocubes was over 90%. Figure 1B shows the size distribution of Pd nanocubes as-prepared, and the size of Pd nanocubes varied from 20 nm to 110 nm, with an average edge length of 49.2 nm.

**Figure 2:**
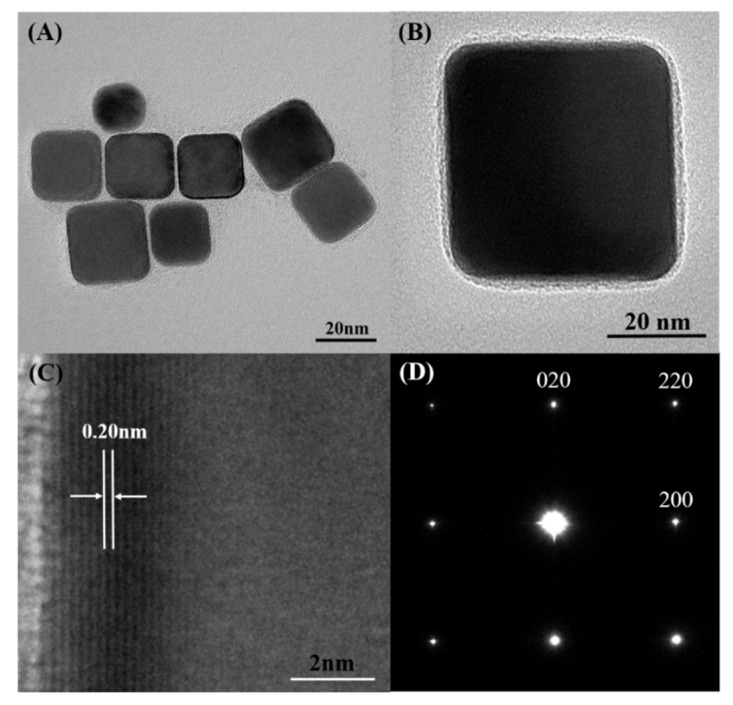
Low-(**A**) and high-magnification (**B**) TEM images of the Pd nanocubes prepared using standard synthesis, (**C**) High-resolution TEM (HRTEM) image of an individual Pd nanocube, (**D**) Selected-area-electron diffraction (SAED) patterns of an individual Pd nanocube.

Figure 2A,B shows the representative TEM images of the Pd nanocubes as-prepared as well as the cubic morphology of the Pd nanoparticles from the low-magnification TEM images. The high-magnification TEM image further confirms that the Pd nanoparticles had perfect cubic shape. Figure 2C shows the High-resolution TEM (HRTEM) image of the individual Pd nanocube seen in Figure 2B, demonstrating clear and consistent lattice fringes on the Pd nanocube. The lattice fringe spacing on both sides of the parallel Pd nanocube was 0.20 nm, which was the (200) interplanar spacing corresponding to the face-centered cubic Pd. Figure 2D shows the Selected-area-electron diffraction (SAED) patterns of the selected individual Pd nanocube, observable along the (001) zone axis. Diffraction points present a perfect square, which indicate that the Pd nanoparticle was a single crystal, enclosed by (100) facets.

To investigate the necessary factors for successful synthesis of Pd nanocubes, we designed and conducted a series of comparative experiments. Previous studies have shown that halogen ions, such as chloride, bromide, and iodide, can be used as effective shape-controlled agents for a shape-controlled synthesis of precious metal nanomaterials in solution phases. These small agents can selectively adsorb on a specific set of crystal surfaces of the nanocrystals during the crystal growth stage. They can change the surface energy of these crystal facets and stabilize the crystal surfaces, therefore preventing the crystal facets from disappearing during the growth of the nanocrystals [27,28]. In this work, we also checked the effect of iodide and compared it with chloride and bromide. Through a series of comparative experiments, we found that, under the current synthesis system, the selection of appropriate amount of iodide ions was one of the key factors for the formation of high-quality Pd nanocubes. If no iodide was added to the standard system for the synthesis of Pd nanocubes, we were unable to synthesize uniformly dispersed Pd nanocubes, instead obtaining Pd nanoparticles with irregular shape and size, as shown in Figure S1. If the other conditions remained the same, we were also unable to obtain uniformly dispersed Pd nanocubes, instead obtaining Pd nanoparticles with irregular size and no selectivity to a specific shape, particularly when only iodide ions were replaced with chloride or bromide ions, as shown in Figure S2. Through this set of controlled experiments, it can be seen that, under the current reaction system, iodide ion is more favorable than chloride and bromide ions to the formation of Pd nanoparticle with cubic morphology. This could be related to the difference in the adsorption strength of halide ions on the surface of Pd nanocrystals, i.e., the adsorption intensity of iodide ions is stronger than that of bromide ions and chloride ions [29]. Chloride and bromide ions could not effectively adsorb on a specific set of crystal facets due to their relatively weak adsorption strength on the surface of Pd nanoparticles in comparison with the iodide ion, and, accordingly, could not stabilize these crystal faces in the growth process of Pd nanoparticles. Therefore, Pd nanoparticles with no selectivity to a specific shape formed under the reaction system involving both chloride and bromide ions. On the other hand, iodine ions could selectively adsorb on the Pd nanoparticle (100) crystal plane and stabilize the Pd (100) crystal plane, leading to the formation of a cubic shape of Pd nanoparticles. These results indicate that halogen ions play an important role in regulating the morphology and size of noble metal nanoparticles, which is consistent with previous studies [30,31]. To check the effect of iodide, we studied the resulting behavior when different amounts sodium iodide were added to the synthesized system. The amount of sodium iodide changed from 300 mg either to 150 mg or to 600 mg; all other conditions remained the same. Results are shown in Figure S3. This demonstrates that, when the amount of sodium iodide was reduced from 300 mg to 150 mg, Pd nanoparticles with mixed morphology, such as cubes, nanowires, tetrahedra and other polycrystalline structures, could be obtained, as shown in Figure S3A. When the amount of sodium iodide was increased from 300 mg to 600 mg, the Pd nanoparticles with no selectivity for a specific shape could form, as shown in Figure S3B. These results indicate that it was necessary to employ appropriate amount of halides for shape-controlled synthesis of nanocrystals in a specific reaction system.

We also investigated the effect of PVP, which was used as a mild reducing agent in the reaction system and whose reducibility resulted from the hydroxyl group located at both ends of the PVP molecule [32]. As shown in Figure S4, when the amount of PVP was reduced from 400 mg to 250 mg, the products could contain cubic, tetrahedral, as well as many other small-size nanoparticles. On the other hand, when the amount of PVP increased from 400 mg to 550 mg, the products were composed of nanorods, tetrahedrons, and polycrystalline nanoparticles. In the current reaction system, PVP largely acts as the reducing agent; the amount of PVP determines the reduction rate of the system, and proper reduction rate is essential for the synthesis of high quality nanocrystals [22]. The results show that an appropriate amount of PVP plays an important role in the formation of high-quality Pd nanocubes.

**Figure 3:**
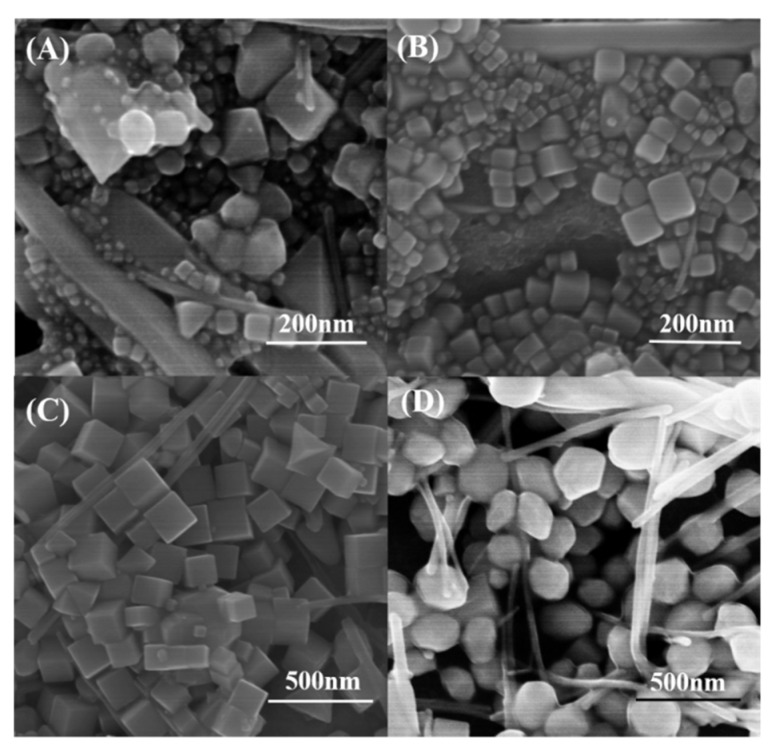
SEM images of the samples obtained at various reaction times for a standard Pd nanocubes synthesis: (**A**) 4.0, (**B**) 5.0, (**C**) 9.0, and (**D**) 15 h, respectively.

To understand the morphological evolution of Pd nanocubes, time sequential evolution experiments were carried out during the hydrothermal process. As shown in Figure 3, at the 4-h reaction stage, the products contained a small amount of cubes; however, most of the Pd nanoparticles had mixed morphologies. When the reaction time reached 5 h, most of the Pd nanoparticles exhibited a cubic shape. When the reaction time was extended to 9 h, Pd nanoparticles continued to exhibit cubic morphology. However, when the reaction time was extended to 15 h, almost all products evolved from cubes to polycrystalline nanoparticles. These results indicate that, in the current synthesis system, the appropriate reaction time was important to obtain cubic nanoparticles. In most cases, after a long period of reaction, it was more likely to obtain thermodynamically favored products, which led to the Ostwald ripening process [33]. We also checked the effect of the reaction temperature. As shown in Figure S5A, when the reaction temperature was reduced to 170 °C, the products were predominantly nanowires and contained a small amount of nanocubes. When the temperature of the reaction system was raised to 230 °C, the products consisted of nanocubes, nanorods, and polycrystalline nanoparticles with irregular morphology as shown in Figure S5B. Through the temperature contrast experiment, we determined that the reaction rate of the system slowed down with the decrease of reaction temperature, which was beneficial to the formation of Pd nanowires. However, with an increase of reaction temperature, the reaction rate of the system was greatly accelerated and the ripening process occurred earlier. Therefore, through temperature contrast experiments, it was found that appropriate temperature control was important for the synthesis of high-quality Pd nanocubes.

In addition, to obtain high-quality Pd nanocubes, we introduced a large number of PVP and iodine ions into the synthesis, which strongly adsorbed on the surface of Pd nanocatalysts, resulting in the occupancy of active sites on the surface of the catalyst and hindering the effective use of catalytic performance. To obtain a clean Pd catalyst surface, we introduced NaBH_4_ treatment, which exploited the ability of Pd to form hydride in the presence of NaBH_4_, efficiently removing capping agents and stabilizers on the surface. The unique ability of Pd surface to form hydrides weakens the interaction between the catalyst and any adsorbed impurities, thus facilitating their easy removal from the Pd surface. Accordingly, a cleaner surface can be obtained without destroying the surface structure of the catalyst, ensuring that the performance of the catalyst can be effectively exerted [26]. As shown in Figure S7, we compared the electrochemical properties of Pd nanocubes in 0.1 M HClO_4_ before and after NaBH_4_ treatment. Following NaBH_4_ treatment, the hydrogen under potential deposition (Hupd) on Pd nanoparticles was obvious; in contrast, before the treatment, this behavior was not clear in the same potential region due to the adsorption of PVP. The surface of Pd nanocubes treated with NaBH_4_ was released. Through our series of comparative experiments, it was found that NaBH_4_ could be effectively used to remove inorganic covers (Cl^−^, Br^−^, I^−^) and organic stabilizers (PVP) from the surface of Pd nanoparticles (Figure S8). After NaBH_4_ treatment, the shape and size of the cubic Pd nanoparticles remained, if slightly agglomerated; however, the signal of PVP and I^−^ could not be found, as shown in Figure S9, which demonstrated the successful removal of these species.

**Figure 4:**
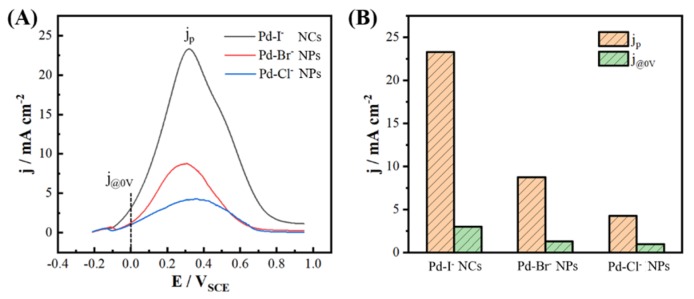
Comparison of electrocatalytic activity of the Pd nanoparticles as synthesized. Pd-I^−^ NCs (Pd nanocubes prepared using the standard synthesis, as shown in Figure 1A), Pd-Cl^−^ NPs (as shown in Figure S2A), and Pd-Br^−^ NPs (as shown in Figure S2B) towards formic acid oxidation. (**A**) Linear sweep voltammetry (LSV) recorded at 50 mV s^−1^ in 0.25 M HCOOH + 0.25 M HClO_4_. Currents were normalized to the electrochemical surface area of the catalysts. (**B**) Comparison of the oxidation current densities at the peak potential (*j*_p_) and at 0 V (*j*_0V_).

To evaluate the electrochemical performance of the Pd nanoparticles as prepared, electrochemical tests of formic acid oxidation were carried out. We investigated the performance of Pd nanocatalyst at 0 V (typical operating voltage of direct formic acid fuel cells) [34,35]. For comparison, the nanoparticles synthesized in the presence of Cl^−^ and Br^−^, respectively defined as Pd-Cl^−^ NPs (Figure S2A) and Pd-Br^−^ NPs (Figure S2B), were used as reference. Figure 4A shows the comparison of the linear sweep voltammetry (LSV) curves for the electro-oxidation of formic acid on the Pd-I^−^ NCs (Pd nanocubes prepared using the standard synthesis, Figure 1A), Pd-Cl^−^ NPs, and Pd-Br^−^ NPs in 0.25 M HCOOH + 0.25 M HClO_4_ solution from −0.21 to 0.95 V at a scan rate of 50 mV s^−1^. The oxidation currents were normalized to the electrochemically active surface area (ECSA), which was measured by integrating the hydrogen underpotential deposition (Hupd) area, as shown in Figure S6. The catalytic activity of Pd nanocubes was significantly superior to those of Pd-Br^−^ NPs and Pd-Cl^−^ NPs. The peak current densities of formic acid oxidation were 23.29, 8.77, and 4.27 mA cm^−2^ on the Pd nanocubes, Pd-Br^−^ NPs, and Pd-Cl^−^ NPs, respectively. The catalytic activity of the cubic Pd nanoparticles, as synthesized, was about twice that of commercial Pd black (11.3 mA cm^−2^) as reported [35]. Figure 4B compared the peak current densities (*j*_P_) and the current densities measured at 0 V (*j*_0V_) of formic acid oxidation on different nanaocatalysts. The *j*_0V_ on Pd nanocubes, Pd-Br^−^ NPs and Pd-Cl^−^ NPs were 3.01, 1.32, and 0.99 mA cm^−2^, respectively. The above analysis indicates that Pd nanocubes exhibited better catalytic activity toward formic acid oxidation in comparison with Pd-Br^−^ NPs and Pd-Cl^−^ NPs. Most of the catalytic reactions took place on the surface of noble metal catalysts. By tuning the surface structure of noble metal nanocatalysts, the catalytic activity of the surface atoms could be fundamentally adjusted. Accordingly, the catalytic performance of the catalysts could be optimized. In this work, Pd nanocubes obtained with the assistant of iodide were well enclosed with (100) crystal planes, which was reported to have excellent catalytic activity among the basic plane of Pd single crystal [36]. Therefore, Pd nanocubes exhibited better catalytic activity toward formic acid oxidation in comparison with Pd nanoparticles prepared in the presence of bromide and chloride ions.

**Figure 5:**
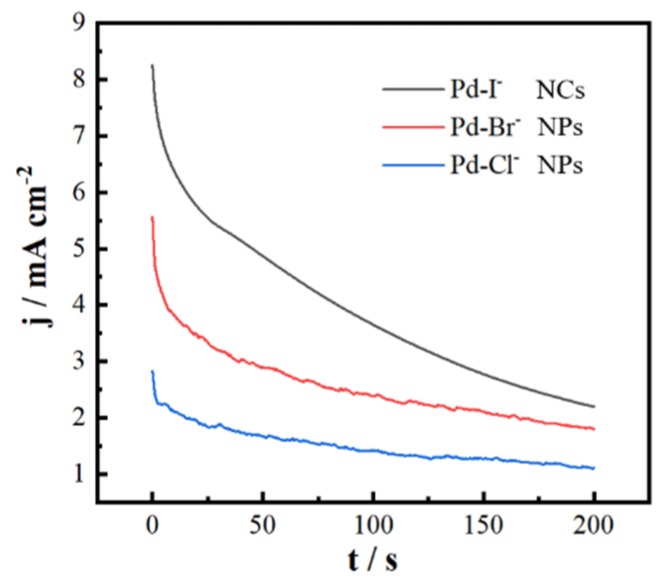
*j*-*t* curves of Pd nanocubes, Pd-Br^−^ NPs, and Pd-Cl^−^ NPs recorded in 0.25 M HCOOH + 0.25 M HClO_4_ at 0.1 V for 200 s.

In addition to the electrochemical activity, the stability of Pd nanocatalysts at 0.1 V was measured using the potentiometric step method. The test time was 200 s; results are shown in Figure 5. The Pd NCs consistently displayed a higher catalytic activity than those of Pd-Br^−^ NPs and Pd-Cl^−^ NPs catalyst over the entire time range of CA measurement. Although the oxidation current on Pd-I- NCs decreased faster than on Pd-Br^−^ NPs and Pd-Cl^−^ NPs, the current density on Pd NCs catalyst maintained a value of 2.23 mA cm^−2^, approximately 1.24 and 2.03 times higher than that of Pd-Br^−^ NPs and Pd-Cl^−^ NPs until 200 s, respectively. The decreases of currents on the nanocatalysts could stem from the consumption of reactants as well as the formation of carbon monoxide, which may have played a role in intermediate poison adsorbing on the surface of catalysts, reducing the likelihood of further reaction on the catalytic active sites.

## 4. Conclusions

We have successfully synthesized high-quality Pd nanocubes through a simple, one-pot method using an aqueous phase. Selection and proper amount of iodide was essential to the successful synthesis of high-quality Pd nanocubes in this synthesis system. When iodide was replaced by other halogens, the Pd nanocrystals with cubic morphology could not be obtained. Parameters such as reaction time, reaction temperature, the amount of PVP, etc. also played an important role in the successful synthesis of Pd NCs. In addition, through our series of comparative experiments, it was found that NaBH_4_ could be effectively used to remove inorganic covers (Cl^−^, Br^−^, I^−^) and organic stabilizers (PVP) from the surface of Pd nanoparticles. Pd nanocubes with clean surfaces exhibited excellent catalytic activity and good stability toward formic acid oxidation.

## Supplementary Materials

The following are available online at https://www.mdpi.com/2079-4991/9/3/375/s1, Figure S1: SEM images of Pd nanoparticles without iodide in the standard system for the synthesis of palladium nanocubes. Figure S2: SEM images of Pd nanoparticles collected from the reactions with the same conditions used in the synthesis of monodispersed Pd nanocubes (Figure 1A) but with I^−^ replaced by (A) Cl^−^ and (B) Br^−^. Figure S3: SEM images of Pd nanoparticles with different amount of sodium iodide (A) 150 mg and (B) 600 mg. Figure S4: SEM images of Pd nanoparticles with different amount of PVP (A) 250 mg and (B) 550 mg. Figure S5: SEM images of Pd nanoparticles with different reaction temperature (A) 170 °C and (B) 230 °C. Figure S6: Cyclic voltammograms of Pd nanoparticles recorded in 0.1 M HClO_4_. Scan rate: 50 mV s^−1^. Figure S7: Cyclic voltammograms of Pd nanocubes (the same batch of samples were treated without NaBH_4_ and treated) recorded in 0.1 M HClO_4_. Scan rate: 50 mV s^−1^. Figure S8: Cyclic voltammograms of Pd nanoparticles (the same batch of samples were treated without NaBH_4_ and treated) (A) Pd-Cl^−^ NPs and (B) Pd-Br^−^ NPs were recorded in 0.1 M HClO_4_. Scan rate: 50 mV s^−1^. Figure S9: (A) The SEM of cubic Pd nanoparticles after NaBH_4_ treatments, (B) Size distribution of the cubic Pd nanoparticles after NaBH_4_ treatments, (C) EDS of the cubic Pd nanoparticles after NaBH_4_ treatments.
