# Supplementary material for: Facile Synthesis of Pd Nanocubes with Assistant of Iodide and Investigation of Their Electrocatalytic Performances Towards Formic Acid Oxidation
Source: Nanomaterials (Basel). 2019 Mar 5;9(3):375. doi: 10.3390/nano9030375 (PMC6474005; doi:10.3390/nano9030375)
Supplement: Supplementary file 1 [file nanomaterials-09-00375-s001.pdf]

Supporting Information

Facile Synthesis of Pd Nanocubes with Assistant of Iodide and Investigation of Their Electrocatalytic Performances Towards Formic Acid Oxidation

Xuan Liu [1,†], Zichao Li [1,2,†], Kuankuan Wang [1], Luming Zhou [1], Xihui Zhao [1], Wenhai Jiang [1], Qun Li [1] and Yujia Deng [1,*]

[1] School of Chemistry and Chemical Engineering, Qingdao University, Qingdao 266071, China; liuxuan2795@163.com (X.L.); zichaoli@qdu.edu.cn (Z.L.); wang13460062584@163.com (K.W.); 2018025207@qdu.edu.cn (L.Z.); zhaoxihui@qdu.edu.cn (X.Z.); jwh1785474676@163.com (W.J.); qunli501@163.com (Q.L.)

[2] College of Life Sciences, Qingdao University, Qingdao 266071, China

* Correspondence: dengyujia@qdu.edu.cn

† These authors contributed equally to this work.

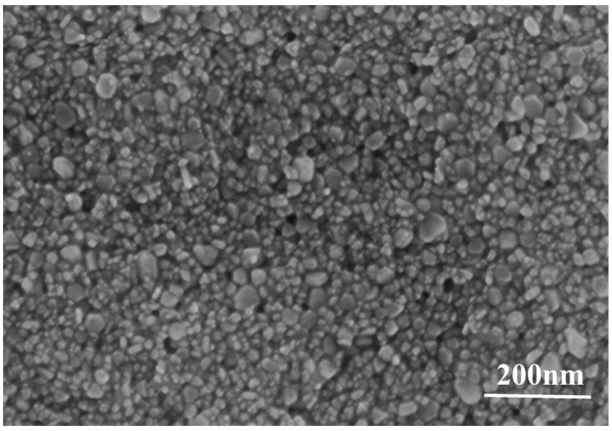

Figure S1. SEM images of Pd nanoparticles without iodide in the standard system for the synthesis of palladium nanocubes.

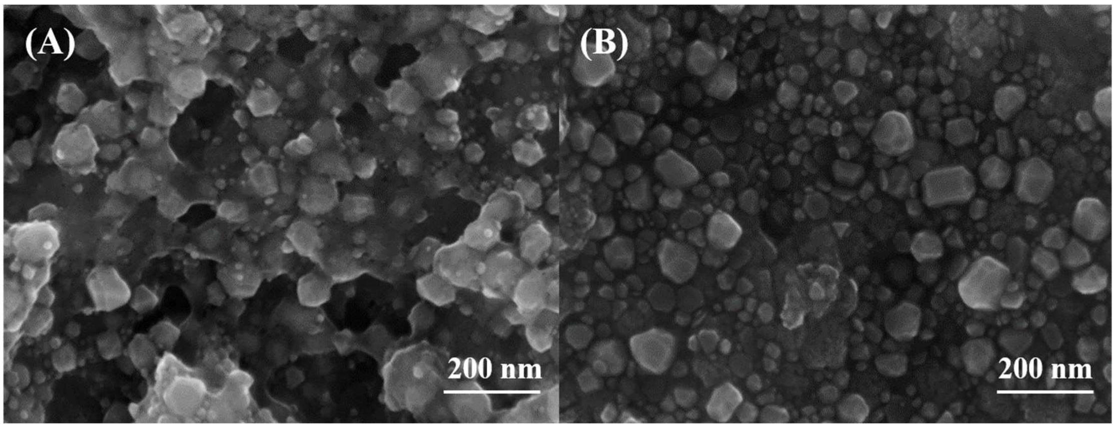

Figure S2. SEM images of Pd nanoparticles collected from the reactions with the same conditions used in the synthesis of monodispersed Pd nanocubes (Figure 1A) but with I^- replaced by (A) Cl^- and (B) Br^-

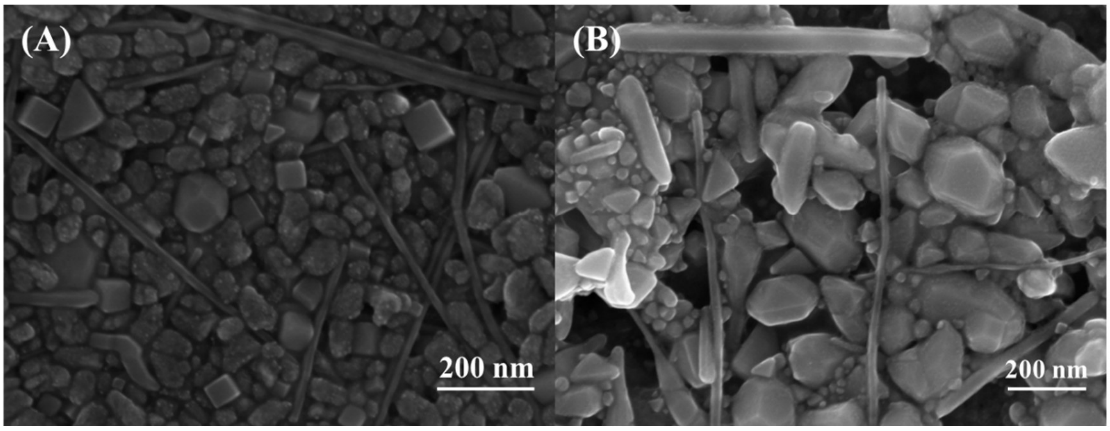

Figure S3. SEM images of Pd nanoparticles with different amount of sodium iodide (A) 150 mg and (B) 600 mg

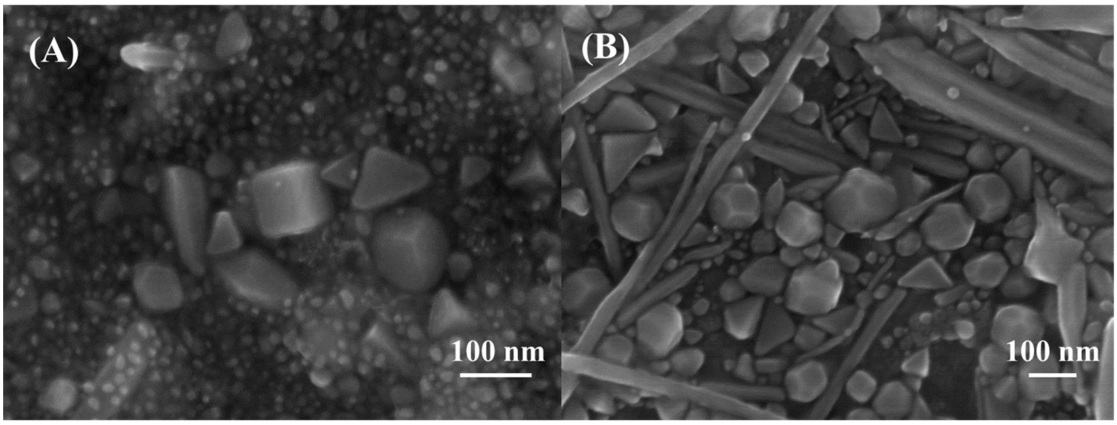

Figure S4. SEM images of Pd nanoparticles with different amount of PVP (A) 250 mg and (B) 550 mg

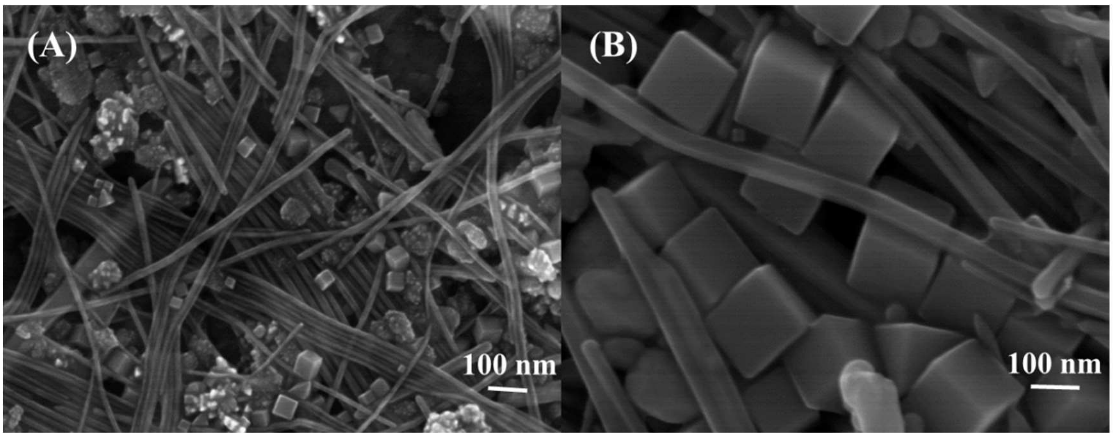

Figure S5. SEM images of Pd nanoparticles with different reaction temperature (A) 170℃ and (B) 230 ℃

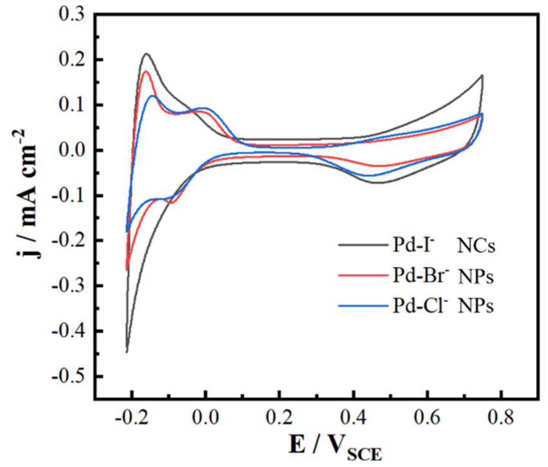

Figure S6. Cyclic voltammograms of Pd nanoparticles recorded in 0.1 M HClO4.Scan rate: 50 mV s^{-1}

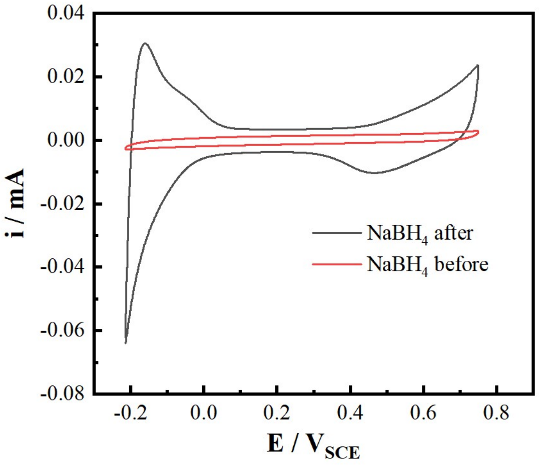

Figure S7. Cyclic voltammograms of Pd nanocubes（the same batch of samples were treated without NaBH4 and treated）recorded in 0.1 M $HClO_4$.Scan rate: 50 mV s^{-1}

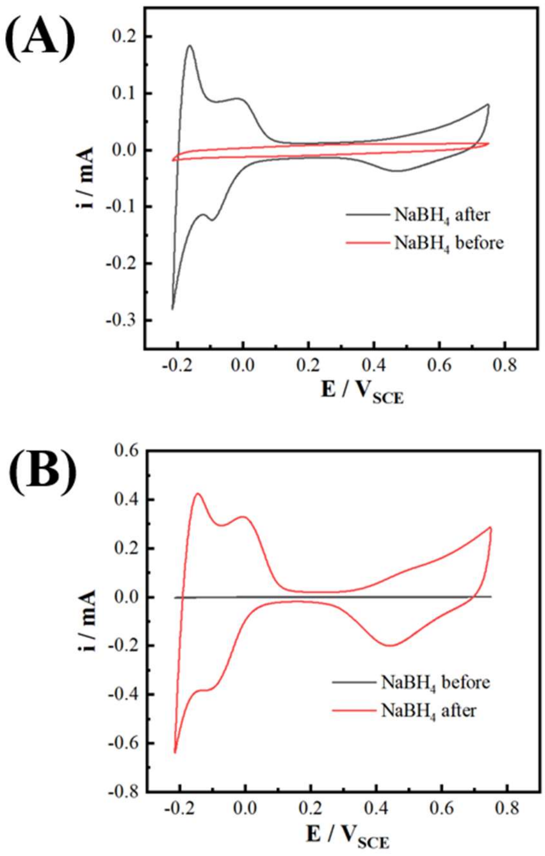

Figure S8. Cyclic voltammograms of Pd nanoparticles（the same batch of samples were treated without $NaBH_4$ and treated）(A) Pd-Cl^- NPs and (B) Pd-Br^- NPs were recorded in 0.1 M $HClO_4$.Scan rate: 50 mV s^{-1}.

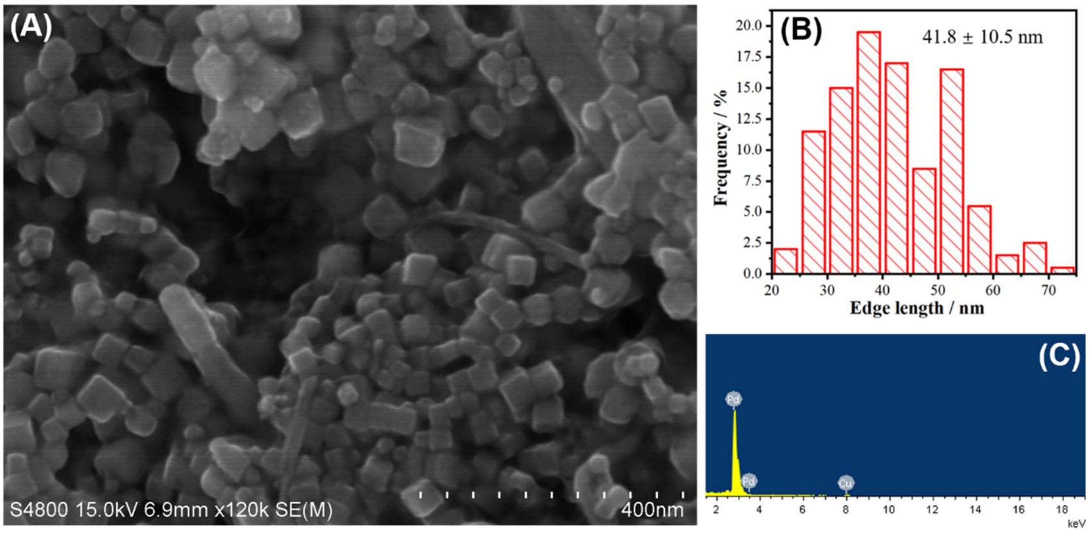

Figure S9. (A) The SEM of cubic Pd nanoparticles after $NaBH_4$ treatments, (B) Size distribution of the cubic Pd nanoparticles after $NaBH_4$ treatments, (C) EDS of the cubic Pd nanoparticles after $NaBH_4$ treatments.
